# From barriers to novel strategies: smarter CAR T therapy hits hard to tumors

**DOI:** 10.3389/fimmu.2023.1203230

**Published:** 2023-07-14

**Authors:** Muhammad Babar Khawar, Fei Ge, Ali Afzal, Haibo Sun

**Affiliations:** ^1^ Institute of Translational Medicine, Medical College, Yangzhou University, Yangzhou, China; ^2^ Jiangsu Key Laboratory of Experimental & Translational Non-Coding RNA Research Yangzhou, Yangzhou, China; ^3^ Applied Molecular Biology and Biomedicine Lab, Department of Zoology, University of Narowal, Narowal, Pakistan; ^4^ Haian Hospital of Traditional Chinese Medicine Affiliated to Nanjing University of Chinese Medicine, Nantong, Jiangsu, China

**Keywords:** CAR T, immunosuppression, immune checkpoints, stromal barrier, transfection strategies

## Abstract

Chimeric antigen receptor (CAR) T cell therapy for solid tumors shows promise, but several hurdles remain. Strategies to overcome barriers such as CAR T therapy-related toxicities (CTT), immunosuppression, and immune checkpoints through research and technology are needed to put the last nail to the coffin and offer hope for previously incurable malignancies. Herein we review current literature and infer novel strategies for the mitigation of CTT while impeding immune suppression, stromal barriers, tumor heterogeneity, on-target/off-tumor toxicities, and better transfection strategies with an emphasis on clinical research and prospects.

## Introduction

Malignant tumors, despite the potency of chemotherapy, remain a resilient adversary and are adept at evading traditional treatment methods, much like a wily fox. Regarding the fact, immunotherapy has been propelled to the vanguard of experimental research owing to its remarkable success in hematological malignancies by utilizing T cells with an expression of chimeric antigen receptors (CARs), albeit solid tumors again outstand owing to cytotoxicity, immunosuppression, heterogenetic nature, an anomaly in metabolism, and resistant tumor territory. Interestingly, CAR T cell therapy appears promising in conquering such obstacles being obliged to nano-immunoengineering (1), gene editing (2), armoring with cytokines (3), dual targeting CARs (4), and switchable CARs (5). The initial development of CARs aimed to augment the efficacy as a single unit; however, later generations focused on the optimization of various aspects of CARs as mentioned earlier. Generally, each generation (as summarized in **Table 1**) has different co-stimulatory domains added, resulting in improved response to tumor cells and longer shelf-life. Exclusively, gen-4-CAR T cells contain constitutive or inducible transgenic sequences to avoid systemic toxicity, and the gen-5-CAR T cells have the addition of an IL-2 receptor for JAK/STAT pathway activation in an antigen-dependent manner. Regardless of such expansion, the actual potential, particularly for solid tumors, is yet to be determined. Moreover, in-depth understandings of the mechanisms of loss of CAR activity, such as CAR T cell exhaustion, antigen escape, and resistance mechanisms, need to be further investigated. Further improvement of transfection strategies is promising to overcome the barriers of the solid tumor microenvironment (TME). Hitherto we have reviewed some novel applications of nanotechnology in immunoengineering of CAR T cells (6), limitations and their possible solutions for improving different generations of CARs (7), and some novel approaches to enhance the efficacy of natural killer cells (NK) with CARs (8). Herein we discuss promising strategies to mitigate CAR T cell-related toxicities. Looking forward, we provide promising insights into the improved delivery of CARs in solid TME and discuss the strategies for better transfection of CAR T cells with an emphasis on translational and clinical research.

**Table 1:** Comparison of the CAR T cell generations.

Generations	Main cytoplasmic domains	Co-stimulatory domain	Drawback
1st generation	A single CD3 ζ-chain	None	Failure to produce adequate IL-2, low cell proliferation, higher toxicities, and brief *in vivo* persistence
2nd generation	CD3 ζ-chain	CD28 for dual signaling	CAR T cell exhaustion
3rd generation	CD3 ζ-chain	CD28 and 4-1BB	Adapted persistence and proliferation yet no enhanced efficacy as compared to earlier generations
4th generation	CD3 ζ-chain	CD28 and IL-12 inducer domain	Off-target/off-tumor toxicity
5th generation	CD3 ζ-chain	Cytoplasmic IL-2 receptor β-chain domain activating antigen-mediated JAK/STAT pathway	Not yet approved Preoccupied with concerns on immunogenicity and high costs

## CAR T cell therapy-related toxicities

CAR T cell therapy-related toxicities (CTT) are owed to various challenges—for example, chance expression of target antigens in non-tumoral cells and heterogenous inflammatory responses (9). The latter fact is duly supported by a recent study which reveal that 79% of patients showed poor prognosis following the CAR T cell therapy, though a fraction of patients showed prolonged remissions upon reinfusion of CARs and salvage treatment (10). In a cohort of 110 patients (NCT03173417), the majority likewise had cytokine release syndrome (CRS) and neurotoxicity (11). In addition, a systematic analysis reported worse CRS (26%) and neurotoxicity (12%) within the cohort, without any significant difference in patients achieving complete remission or those experiencing inflammatory responses (12). Nonetheless, reinfusion of CARs following the failure of prior CAR T cell therapy showed prolonged remission rates (13) and, combined with a high dose of cyclophosphamide, resulted in CAR T cell expansion in peripheral blood (NCT01860937) (14). Apart from this, studies have reported various forms of CTT—for example, hemophagocytic lymphohistiocytosis was observed in approximately 14.8% of pediatric and young adults who underwent CD19-specific CAR T cell therapy (15). Moreover, tumor lysis syndrome (16), hypogammaglobulinemia (17), and febrile neutropenia (NCT02414269) (18) have been reported less frequently in association with CTT. The fact is attributed to several factors, such as the variable nature of targeting antigens and individual patient factors. Additional research is imperative to enhance our comprehension and mitigate the potential risks and CTT beyond CRS and neurotoxicity. A comprehensive exploration of these aspects will enable the refinement and optimization of current strategies, thereby enhancing the safety and efficacy of CAR T cell therapy.

### Mechanism of CAR T cell therapy-related toxicities

Henceforward, two kinds of CTT are common: CRS and neurotoxicity. The exact mechanism of the pathophysiology of CTT is yet to be demarcated. However, studies have identified a significant mechanism involving an interaction between macrophages and T cells, leading to the release of multiple cytokines and oxides in murine models (19). Subsequently, it was suggested that the infusion of CAR T cells following macrophage depletion could be a promising approach to eliminate CRS (20). Although this depletion increases the risk of infections, the complications can be managed using IL-6 or corticosteroids or a combination of both (21). Recently, another study showed a positive correlation between CRS and hematological toxicities (22). Endothelial activation is a potent member in CRS pathology (23) as supported by a cohort of 133 B cell malignant patients who received CD19 CAR T cell infusion in a dose-dependent manner. In total, 70% of patients developed CRS with variable severity indices (24). Not just this—endothelial activation also disrupts the blood–brain barrier (BBB) in patients with neurotoxicity as warranted by the magnetic resonance imaging results of severe cases (23). A mechanistic approach was developed in a study that showed that CAR T cell products such as tisagenlecleucel responded by the expansion of proliferative memory-like CD8 clones, while another product, axicabtagene ciloleucel responders, displayed more heterogeneous populations. The latter non-responders had elevations in CAR T-regulatory cells, which suppressed conventional CAR T cell expansion and drove late relapses in an *in vivo* model (25). One of the recommendations, in the context explained above, is reinfusion of CAR T cells along with a combinatorial use of IL-6 and steroids inclusive of former macrophage depletion. Nevertheless, there are various other mechanisms of pathology of CTT, which are potent to grab the attention of researchers, yet careful monitoring and personalized treatment plans are warranted to manage the potential adverse reactions and ensure the safety of the patients.

### Mitigation of CAR T cell therapy-related toxicities

Currently, some strategies have been reported for the mitigation of CTT; however, further understanding of the exact approach will lead to the precise development of CARs. Researchers are focusing on the preemptive mitigation of CTT in combination with various immunosuppressors which have been proven to be effective devoid of any attenuation of CAR T efficiency—for instance, a clinical study confirms no negative impact on the antitumor activity of CAR T cell therapy when combined with tocilizumab and/or steroid administration and supports no expansion and persistence of CD19-targeted CAR T cells (26). Following the conclusion, a prospective study evaluated the efficacy of tocilizumab to mitigate CRS in 70 patients divided into two groups of high and low tumor burden. The overall response rates (87% in high-tumor-burden and 100% in low-tumor-burden patients) were good enough to support the very strategy as an effective one. The clinical trial successfully met the primary and final goals of the study without any attenuation of the antitumor activity of CAR T cell therapy (27). Dexamethasone—a glucocorticoid, has also been of interest in the mitigation of neurological toxicities due to its exceptional penetration into the central nervous system as per the guidelines of the National Cancer Institute. IL-7 receptor alpha is a well-recognized facet needed for CAR T cell persistence and memory T cell formation (28). In this case, dexamethasone has been shown to upregulate IL-7 receptors to enhance CAR T cell persistence and antitumor activity (29). Urak and colleagues suggest that the effect of dexamethasone is not only restricted to specific T cell subsets while the same upregulation was also observed in peripheral blood mononuclear cells (PBMCs), naïve cells, memory T cell-derived CAR T cells, and untransduced T cells. The study does not affect the efficacy of CAR T cells *in vivo* or *in vitro*. These facts are promising in understanding the mechanism of dexamethasone in CAR T cell therapy. When combined with ruxolitinib, dexamethasone synergistically reduced the disease symptoms in a murine model. Mechanistically, JAK-dependent cytokines IL-2 and IL-12 conferred resistance to dexamethasone, but not etoposide, and ruxolitinib attenuated STAT5 activation to enhance dexamethasone-mediated cell death in the presence of IL-2 or IL-12 (30). Other various drugs are under investigation to be used in combination with CAR T cell therapy. In this text, we advocated the inhibition of CTT utilizing reported cytokines and combining various immunosuppressants and steroids. These drugs reveal an in-depth understanding of the mechanism of mitigation of CTT, including CRS and neurotoxicity. Further research in this area is *de rigueur* to proceed with the mitigation of CTT for effective CAR T cell therapy.

## Improving the barriers for better delivery in tumor microenvironment

Following a pertinent success in hematological malignancies where CAR T cells find fewer disturbances for the effective killing of tumor cells, solid TME presents several physical and immune barriers that impede CAR T cell trafficking in solid TME. These include heterogeneity of tumor antigens, nutrient-poor milieu, T cell exhaustion, and recruitment of immunosuppressor cells.

### Immunosuppression

**Figure 1:**
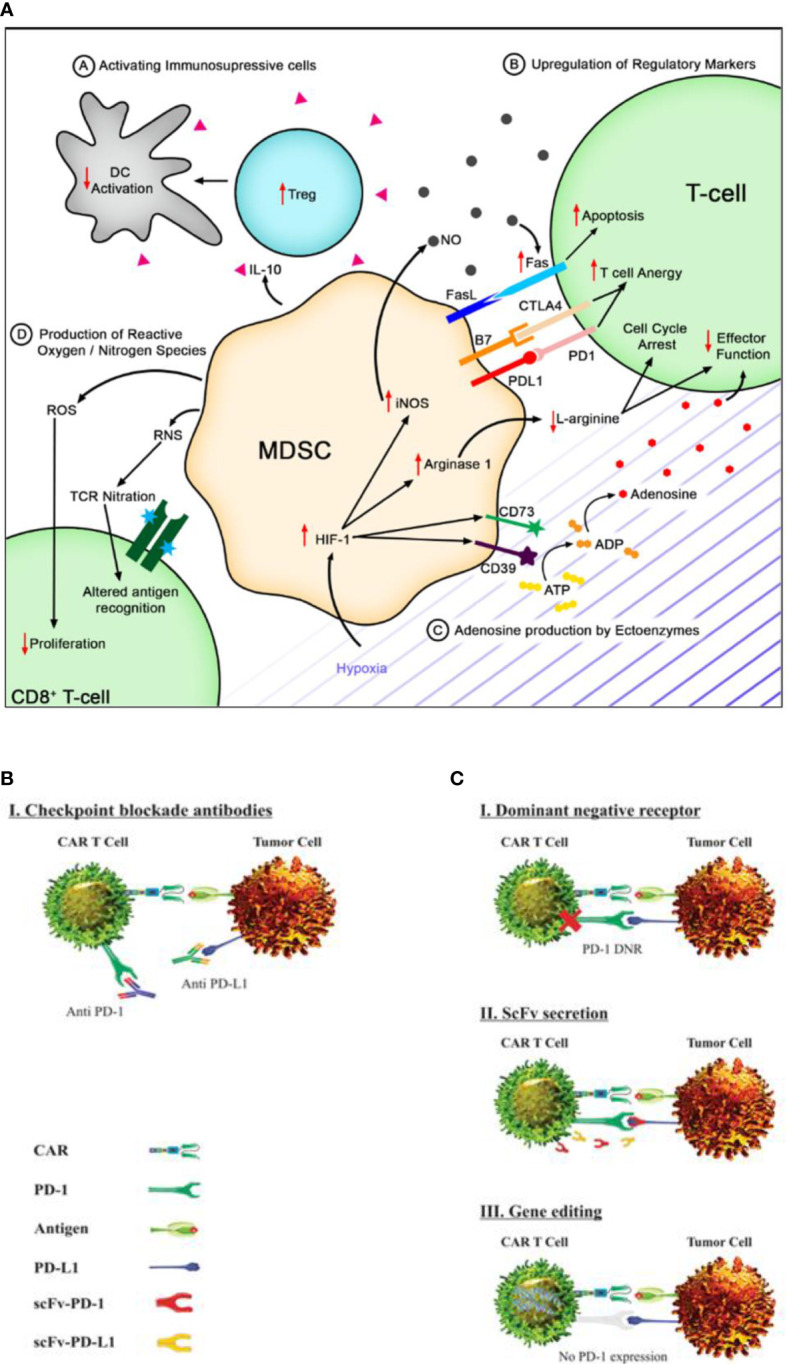
Immune suppression and checkpoint inhibition in solid tumor microenvironment. **(A)** Myeloid-derived suppressor cells (MDSCs) secrete IL-10 to activate immunosuppressive cells like T-regs, leading to reduced T cell activation. They can also induce the upregulation of checkpoint molecules on T cells, inducing anergy or apoptosis. Hypoxia in the tumor microenvironment can upregulate CD73 and CD39, leading to increased adenosine. MDSCs produce ROS and RNS that can decrease T cell proliferation and alter antigen recognition [reproduced from (36)]. **(B)** Immune checkpoint blockade using monoclonal antibodies targeting PD-1 and PD L1 has shown promise in restoring anti-tumor immune responses by rescuing T cell function and enhancing T cell-mediated killing of cancer cells [reproduced from (37)]. **(C)** Genetically engineered chimeric antigen receptor T cells can locally inhibit immune checkpoint molecules by expressing dominant-negative receptors, secreting blocking scFv, or disrupting gene expression to interfere with intracellular signaling [reproduced from (37)].

Mechanistically, chemokine-transforming growth factor β (TGF-β) activates tumor-associated fibroblasts, which, in return, upregulates the extracellular matrix proteins to T cell infiltration into solid TME (31). Interestingly, a direct restriction to CAR T cell infiltration by TGF-β has also been observed, whereby TGF-β not only downregulates CXCR3 expression but also increases the binding of Smad2 to CXCR3 promoter (32). The search for effective and safer therapeutics targeting the TGF-β pathway is further complicated by various functions that TGF-β performs in normal tissues (33). We accordingly advocate further extensive research to investigate the contextual role of TGF-β for immunosuppression in TME. Rather than TGF-β, several other cytokine and chemokine profiles recruit T-reg cells, myeloid-derived suppressor cells (MDSCs), and tumor-associated macrophages and thus limit CAR T cell infiltration. T-reg cells secrete immunosuppressive cytokines and downregulate antigen-presenting cells via cytotoxic T lymphocyte antigen 4, thus preventing T cell activation (34). The immune-suppressive effect of MDSCs also has an unfavorable effect on CAR T cell therapy (35). Krishnamoorthy et al. reviewed a detailed mechanism of suppression of T cells mediated by MDSCs (36). They stated that, generally, MDSCs suppress the immune response by inhibiting the functions of T cells in four distinct pathways as shown in **Figure 1A**. In this way, we recommend utilizing the drug which could be capable of blocking the MDSC functions within the solid TME. In this aspect, the delivery systems must be capable of targeted unloading of cargo. We also suggest that CAR T cells should be engineered genetically to express molecules that specifically target and eliminate MDSCs within the TME. This recommendation is interestingly supported by a study in which the authors developed CAR T cells with co-expression of receptors for MDSCs. The study provides an evidence of superior anti-tumoral activity of CAR T cells which also improved T cell proliferation in solid TME (38).

### Tumor heterogeneity

Once the minute, safe, and appropriate tumor-associated antigens (TAAs) are determined in the development of CAR T cell therapy, it becomes crucial to address tumor antigen heterogeneity in the subsequent step, as it may be expressed variably in both tumor and normal cells (39, 40). Dana H et al., in 2021, systematically summarized various antigens, targeted in different cancers (41), showing promise in the mitigation of tumor heterogeneity. An interesting study employing a combinatorial therapy demonstrated a compatibility between CARs, T cell receptors (TCRs), and hnCD16 in alleviating tumor heterogeneity (42). This technique is likewise effective for resistant tumors owing to antigen escape. By computational modeling, cellular interactions were examined, and it was thus concluded with a suggestion to employ combinatorial therapy for effective treatment (43). As mentioned above in the mitigation of CTT, the presence of IL-12 enhances the synergistic effect of steroid therapy in combination with ruxolitinib. This is an evident boon that it is present in solid TME (44). We hereof recommend a tumor-specific condition employing the IL-12-mediated expression of CARs. Nevertheless, investigations are warranted to inspect off-tumor toxicity along with IL-12 dosages. A similar study was conducted aiming to augment tumor specificity (45). NK cells detect tumor ligands through NK receptors, including NKp30. A subset of CD8+ T cells expressing NKp30 recognizes B7H6, a tumor ligand frequently overexpressed in cancers (46). Apropos of this research, Correia et al. (2021) latterly engineered a dual recognition strategy in the form of innate-like NKp30+CD8+ T cells with a combined expression of TCRs and CARs, thus successfully targeting tumor heterogeneity (47). Furthermore, novel criteria for the conscription of CAR T cell therapy, including preselection for the intensity and proportion of targeted antigen, are warranted. Although prescribing the definite criteria could be tricky, hereto we suggest concurrent or in combination targeting of different tumor antigens, which is promising in leading to a more effective antitumoral effect and possibly reducing the emergence of tumor variants that lack the targeted antigen.

### Immune checkpoint inhibition

Generally, upon tumor initiation, normal and tumor cells upregulate inhibitory signaling pathways, driving the tumor progression and its immune escape. Blockade therapy has shown to reverse this suppression and has shown promising effectiveness in hematological malignancies (48). Henceforth, the use of immune checkpoint inhibitors, specifically PD-1 blockade, has been under investigation. Chronic antigen stimulation in tumor sites leads to inhibitory receptor upregulation and CAR T cell exhaustion in a PDL1-dependent manner (49). Blocking the PD-1:PDL1 interaction using mAbs can rescue exhausted CAR T cells as shown in **Figure 1B**, but multiple administrations are required due to the short half-life of antibodies and immune-related adverse events (50). Localized immune checkpoint inhibition at the tumor site using modified CAR T cells secreting PD-1 blocking scFv can increase the killing activity and restore the cytotoxic activity in solid tumors. The very strategy has shown a positive response in CAR T cell exhaustion *in vivo* that indicates a promising synergistic effect with improved efficacy (51–53). Genetically modified CAR T cells are also under investigation for local inhibition of immune checkpoints incorporating specific mechanisms—for example, engineered CAR T cells expressing a dominant-negative receptor can disrupt the signaling of immune checkpoint molecules. Genetically modified CAR T cells also secrete blocking single-chain variable fragments, which can prevent immune checkpoint molecules from binding to their targets. Lastly, CAR T cells can be engineered to interfere with intracellular signaling pathways by disrupting the gene expression as depicted in **Figure 1C** (54). By implementing these innovative techniques, it is anticipated that CAR T cell therapy can overcome immune checkpoint-mediated suppression and offers enhanced therapeutic outcomes for cancer patients.

MDSCs can upregulate checkpoint molecules like CTLA4, T cell immunoglobulin, and mucin domain-containing protein 3 (TIM3) on T cells, leading to T cell apoptosis through Fas signaling (55). Another study focuses on targeting B7-H3 immune checkpoint in non-small cell lung cancer which shows promise in T cell activation and target cell death. The study also finds that anti-B7-H3 blockade has a role in altering glucose metabolism through reactive oxygen species-mediated pathways (56). Moreover, rebalancing the TGF-β1/BMP signaling pathways shows promise in exhausted T cells and induces higher antitumoral targeting while synergizing the immune checkpoint blockade (57). This data may provide better insights into solid TME for effective tumor-killing activity.

### On-target/off-tumor toxicities

A careful selection of tumor-specific antigens is warranted as they are rare and require precise identification following the use of TAAs. The main issue is the heterogeneity of tumor-specific antigens and their expression on normal, non-tumoral cells vulnerating them for off-tumor toxicity. Generally, the antigens targeted by CAR T cells are tumor-associated, rather than tumor specific. While these antigens may be highly expressed within the solid tumor microenvironment, it is important to note that normal cells also express a small amount of these antigens (58).

Recent developments in the field of CAR T cell therapy have led to the emergence of novel technologies which hold significant potential in addressing the challenges of on-target/off-tumor toxicity. Researchers have developed novel technologies like ZAP-70-based logic-gated intracellular network CARs through the application of Boolean logic gating and the engineering of intracellular T cell signaling molecules (59). This fact demonstrates superior efficacy and safety while bypassing upstream signaling proteins. The utilization of logic gating circuits and synthetic biology in CAR T cell engineering has shown promising specificity in preclinical mouse models (60). However, the translation of these approaches into clinical studies is yet to be fully explored and evaluated. Furthermore, SynNotch CAR utilizes synthetic Notch receptors in mitigating the toxicity by enhancing the specificity and functionality (61). It allows enhanced selectivity via improved discrimination between tumor cells and healthy tissues, minimizing off-target toxicity (62). The activation of SynNotch CAR is likewise programmable by enabling downstream signaling pathways and expressing effector molecules to offer enhanced flexibility (63). Notably, it also allows fine-tuning of immune responses by incorporating inducible signaling domains or regulatory elements that modulate CAR-T cell activity, promoting controlled and adaptable immune responses (64). Another important development is the T cell redirected for universal cytokine killing (TRUCK) CAR which combines the killing ability of CAR with the production of therapeutic cytokines (65) such as inducible IL-18 (66). There are conditional cytokine expression systems that enable controlled cytokine release, reducing the risk of off-target effects and cytokine-related toxicities (67, 68). Importantly, TRUCK CARs allow antigen-specific targeting minimizing off-target toxicity (69, 70).

**Figure 2:**
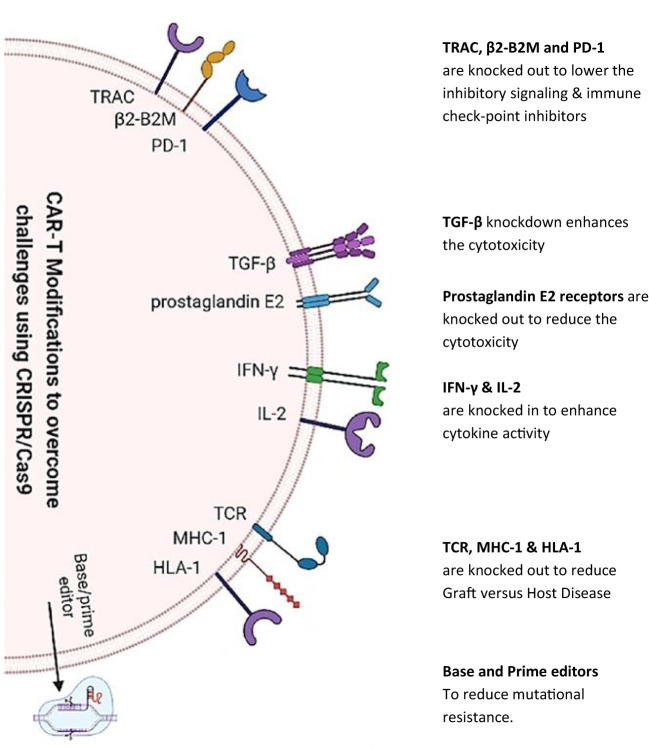
CRISPR/Cas9 knock in/out of various targeted genes to modify chimeric antigen receptor T cell therapy. CRISPR/Cas9 technology [as depicted by Khan et al. (2022)] is being utilized to reduce inhibitory signaling, knock in/out genes for toxicity reduction, and boost cytokine secretion. Furthermore, gene editing has been used to reduce adverse host reactions by knocking out TCR, major histocompatibility complex class 1 (MHC-1), and HLA-1 genes.

Specifically, apart from the above-mentioned developments, gene editing practice is more promising in this research area. To enhance T cell responses to antigens and decrease exhaustion rates, the very technology has been employed (71). Furthermore, the use of such technologies may allow for the development of T cell populations that can be used in allogeneic applications. Exploiting clustered regularly interspaced short palindromic repeat (CRISPR) in mitigating on-target/off-tumor toxicities is itself another research arena whereby it is used to create CAR resistance to immune suppression (72). Modifying CAR T cells to target tumor-specific antigens using CRISPR is another promising strategy as it has been demonstrated that CRISPR has been used to knock out the expression of the CD19 antigen on normal B cells, allowing CAR T cells to target only tumor cells that express CD19 (72). This approach has the potential to reduce the risk of CTT without targeting normal cells. An extensive review of such modifications via CRISPR technology for CARs (as shown in **Figure 2**) presents encouraging outcomes in clinical and preclinical trials (73). Further research into all these insights is necessary to develop effective strategies for mitigating the risks associated with CAR T cell therapy.

### Tumor stromal barrier

**Figure 3:**
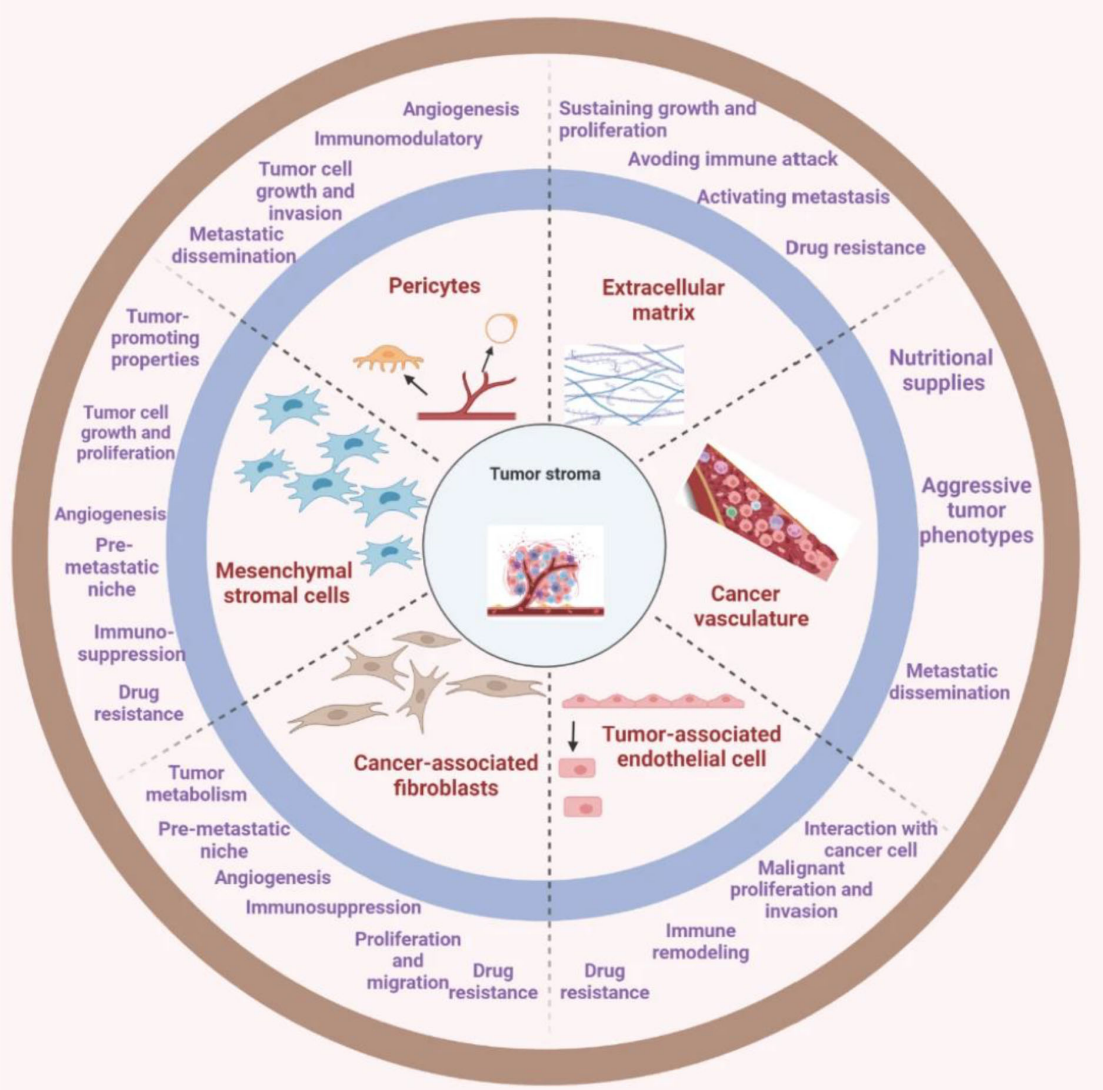
The tumor stroma comprises various key entities. The deep understandings present a comprehensive mechanism of the interactions between tumor and stromal cells and how they contribute to the development and progression of cancer. This can be crucial in the development of targeted therapies that specifically address the tumor microenvironment to improve treatment outcomes for cancer patients [reproduced from (75)].

Herein the aforementioned barriers have been on the bull’s eye to steer CARs toward promising efficacy. However, newly emergent evidence suggests novel roadblocks in the form of tumor parenchyma and stromal cells in solid TME (74). These two distinct yet synergistic elements engage in a dynamic cross-talk, thereby facilitating and sustaining a resistant solid TME. Notwithstanding, the very notion not only initiates and promotes tumor growth but also augments the metastatic potential of tumors (75), presenting new research areas in overcoming the roadblocks to CAR T cell therapy. The promising candidates comprise tumor-associated fibroblasts (TAFs) secreting fibronectin and collagen-like proteins. Further cellular entities such as MDSCs, epithelial, endothelial, or mesenchymal cells, adipose cells, and bone marrow-derived mesenchymal cells have also been observed to transit to TAFs (75, 76) as depicted in **Figure 3**. Noticing all these entities, CAR T cell therapy acquires a giant barrier to incapacitate. Consequently, the development of innovative strategies and combinatorial strategies with other established treatment modalities are warranted as a promising gateway to CAR T cell therapy research arena.

Accordingly, modulation of TME in overcoming stromal barriers has shown promise. Geng F. et al., in 2019, examined the effectiveness of a DNA vaccine expressing fibroblast activation protein-α (FAP-α) in altering the solid TME in breast cancer (77). In this aspect, CARs that should target FAP-α to mitigate the barrier are needed to be developed, allowing more infiltration and persistence, or else CAR T cell therapy may be combined with the pre-infusion of nanoparticles (NPs) with FAP-specific antibodies cargo as evidenced by Zhen et al. in 2017 (78). There are a lot more options; one of that is targeting pathways of stromal interactions which may be employed as a pre-emptive strategy or combinatorial agents as extensively reviewed by van der Spek et al. in 2020 (76).

By and large, the text herein highlights the potential of CAR T cell therapy and underscores a prerequisite for sustained research and collaboration to optimize its clinical application and offer hope for patients with solid tumors.

## Better transfection strategies in the development of CAR T cells

There is a multitude of methods for transfecting the CAR T gene, most notably electroporation, viral transduction to RNA electroporation, and even newer means of improving the overall efficiency of CAR T cell therapies, such as the manipulation genes which have produced valuable desired nucleases: transcription activator-like effector nucleases (TALEN) and the widely known CRISPR (79).

However, even with the large success and even more so the novelty, there are many limitations to its administration. As mentioned earlier, electroporation, one of the most commonly used means of transfection—has shown to have many limitations: non-specific delivery and undesired targets, accompanied with certain cell death rates (80). Studies have also indicated a risk of cytotoxicity (81). The transfection through electroporation correspondently results in membrane disruption that affects expressions. Another limitation faced by electroporation is its inadequacy in its application for *in vivo* purposes (82).

These limitations have been and can be tackled in many ways, one being by incorporating conventional anti-cancer drugs (83). The findings suggest approaching certain targeted tumors by combining specific mathematical and statistical computer models that have shown to improve the desired result and efficacy of transfecting CAR T through electroporation (84, 85). Perfusing the targeted organ or tissue or the surrounding vessels could also enhance the outcome. A study suggests that CAR Ts may be produced via plasmids and IL15-IL15R fusion proteins, resulting in effectiveness and superior endurance in *in vivo* environments (86).

CAR T cells are often challenged in forming proper resistance, of which the expression of the desired targeted antigen has been shown to be either partly or completely lost by many tumors (87). Several studies have indicated that certain undesired downregulation of expression was observed. With the viral transfection methods, the limitations are more inclined to expense viability. The production of high-grade and clinically safe viral CAR T cells is quite expensive (88). With that, the general viral CAR T cells are highly complex structures and require intense observations in their completion. The prolonged expression of these CARs has led to tonic signaling and cell death via activation induction of T cells. Even in the case of the rather novel CARs, such as the aforementioned TALEN, the authors discussed how receptor downregulation may mute the activation of natural killer cells caused by the lack of MHC-I (89, 90).

As mentioned earlier, another prevailing setback of CAR T cell therapy may result in neurotoxicity, which is a result of the CAR T cells prompting CRS, which has been observed in many patients treated with CAR T cell therapy (91). A way to improve this setback is by developing a method that can alter the switching ability of CAR T cells, and we advocate that switchable CARs may provide temporal control over neurotoxicity and effector performance. When toxicity first manifests, turning down the CAR T cell effector activity with medication can be helpful and life-saving. However, after the toxicities are gone, they will need to be switched back on to prevent impairing the antitumor activity. This is well elaborated by a recent study via the use of protease-regulated signal neutralization by an inhibitable protease (SNIP) (92). Comparatively to constitutively active CAR T cells, SNIP CAR T cells displayed higher cytotoxicity against tumors, however an *in vivo* durability and more likely a memory phenotype. The expression of markers linked to T cell exhaustion was significantly reduced in SNIP CAR T cells, perhaps as a result of avoiding prolonged T cell activation.

Lastly, the overall cost of CAR T has oftentimes been the issue in developing and improving the therapy. Hence, to lower the overall cost of CAR T cell therapy, better and more affordable gene transfer methods are required. Although lentiviral/retroviral vector-mediated gene transfer is more expensive than non-viral methods, there is a growing body of clinical data supporting the latter method (93).

## Translational and clinical research

Notwithstanding significant clinical successes, there remain challenges in the translation of CAR T cell therapy to routine clinical practice. Currently, CTT mitigation, improving barriers for better trafficking into the TME, and transfection approaches are of major focus in clinical research as shown in **Table 2**. A preclinical study focusing on checkpoint blockade demonstrated that the co-expression of PD-1-CD28 in TRuC T cells enhances cytokine production and supports anti-tumor efficacy in xenograft mouse models. The results suggest that PD-1-CD28 co-expression could have therapeutic potential in preventing PD-L1-induced T cell hypofunction (94).

**Table 2:** Summary of CAR T cell therapeutic approaches to clinical research in improving the barriers.

Sr.	Focus	Disease	Status	Registration number
CRISPR gene editing technology				
1.	CD19-specific CAR T cells with edited endogenous HPK1	CD19+ leukemia or lymphoma	Recruiting	NCT04037566
2.	Mesothelin-directed CAR T cells by knocking out PD-1 and TCR gene	Multiple solid tumors	Unknown	NCT03545815
3.	TCRendo and PD-1 edited T cells	Melanoma	Terminated	NCT03399448
4.	Allogeneic anti-CD19 CAR T cell therapy	Lymphoma	Recruiting	NCT04637763
Mitigation of CAR T cell therapy-related toxicities				
5.	Preemptive mitigation using siltuximab with CD19-CAR T cell therapy	Non-Hodgkin lymphoma	Recruiting	NCT05665725
6.	Autologous mesothelin-targeted CAR T cells secreting PD-1 nanobodies	Solid tumors	Phase I/recruiting	NCT05373147
7.	Mucin1 cell surface-associated C-terminal CAR T cells	Multiple solid tumors	Early phase I/recruiting	NCT05239143
8.	Anti-B-cell maturation antigen T cells	Multiple myeloma	Completed	NCT02215967
Improving the efficacy of CARs with other strategies				
9.	EGFR806 CAR T Cell immunotherapy	Multiple solid tumors	Phase I/recruiting	NCT03618381
10.	LCAR-M23 CAR T cell therapy	Epithelial ovarian cancer	Terminated	NCT04562298
11.	Lentivirus-transduced CAR T cells	Multiple solid tumors	Active, not recruiting	NCT03302403

Tumor-specific antigens have also been targeted in several clinical trials reducing off-tumor toxicities—for instance, NCT04489862 is an early phase I study which aims to target α-PD-1 and MSLN in non-small cell lung cancer at Wuhan Union Hospital, China. Another phase I study (NCT03198052) is recruiting patients. The study aims to target various antigens such as mesothelin, MUC1, PD-L1, and EGFR. Several studies have successfully obtained higher response rates for solid tumors using monodrug synergized with CAR T cell therapy—for example, a study (NCT03548207) enrolled 97 patients and used cyclophosphamide and fludarabine as an autologous CAR T cell monodrug and obtained a 95% overall response rate.

For CRISPR technology in CAR T cell therapy, there are a limited number of clinical studies for solid malignancies—for instance, phase I study (NCT03545815) establishes a groundwork utilizing PD-1 and TCR disruption for solid TME (95). None of the participants displayed any unexpected adverse events or dose-limiting toxicity. The post-infusion detection of predominantly TCR-positive CAR T cells was observed in the peripheral blood of merely three patients. Preclinical experiments suggested a decrease in the persistence of TCR-deficient CAR T cells, encouraging further investigation in human clinical trials. Various other clinical studies such as NCT04037566 are currently underway to investigate the effectiveness of CD19-CAR T cells modified with endogenous HPK1 for treating lymphoma.

## Conclusion and future prospects

CAR T cell therapy for solid TME is presenting encouraging results subsequent to its success in hematological malignancies. The incapacitation of various barriers for CAR T cells is under extensive investigation to achieve therapy with mitigated CTT, improved delivery to solid TME impeding blockades, and better transfection strategies. While improved outcomes have been observed, CARs still face numerous hurdles that need to be overcome. In this way, this review presents strong recommendations and suggestions highlighting the gaps and areas of focus. There is an urgent need to focus on immunosuppression due to cytokines and immunosuppressive cells. Investigations for CAR T reinfusion with the combination of cytokine/steroids or other immunosuppressors for preemptive mitigation of toxicities are warranted. While immune suppression has been observed with chemokine inhibition, such as TGF-β, the beneficial effects of these molecules should not be overlooked under normal circumstances. Therefore, we recommend comprehensive research in this area, taking into account their contextual roles. Additionally, CRISPR technology can effectively overcome immune checkpoint inhibition and other barriers.

Even though significant progress has been made in the development of CAR T cell therapy, further research is required to address the remaining challenges and optimize its clinical application. With ongoing advancements and collaborations in the field, CAR T cell therapy may provide new hope for patients with previously incurable malignancies. Finally, improvements in CAR T cell therapy *vis*-*à*-*vis* to herewith discussed prospects may prove to be promising in refining the current research in fighting against solid tumors, which can steer CARs toward another all-embracing victory in solid tumors.

## Author contributions

MK, FG and AA proposed the idea, collected the data, and wrote the manuscript. HS proposed the idea and modified, supervised, and approved the final version of the manuscript. All authors contributed to the article and approved the submitted version.
